# Book Reviews

**Published:** 1903-03

**Authors:** 


### BOOK REVIEWS.

Medical Epitome Series. Diseases of the Skin. A Manual for Students and Practitioners. By Alfred Schalek, M.D., Instructor in Dermatology, Rush Medical College, Chicago, Ill. Lea Bros. & Co., New York and Philadelphia, 1903.

This little book contains concisely stated the elementary facts of dermatology. It is not intended as a text-book, but is designed to give in outline the essentials of this branch of medicine. The illustrations are good, the scope of the book comprehensive and the purpose of it seems well accomplished. It will fit very well for the needs of the student, and it may be recommended to him with confidence.

---

The Mattison Method in Morphinism. A Modern and Humane Treatment of the Morphine Disease. By J. B. Mattison, M.D., Brooklyn, N. Y. E. B. Treat & Co., Publishers, New York. Price $1.00.

The author, who is well known in connection with the treatment of drug addiction, after thirty years of experience gives the result of this experience. He advocates the use of sodium bromide with moderately rapid withdrawal of the drug. The additional treatment is hygienic, moral and symptomatic. The brochure should be read by all who are interested in the therapy of drug habits.

